# Reducing Breast Cancer Recurrence: The Role of Dietary Polyphenolics

**DOI:** 10.3390/nu8090547

**Published:** 2016-09-06

**Authors:** Andrea J. Braakhuis, Peta Campion, Karen S. Bishop

**Affiliations:** 1 Discipline of Nutrition and Dietetics, FM & HS, University of Auckland, Private Bag 92019, Auckland 1142, New Zealand; pcam131@aucklanduni.ac.nz; 2 Auckland Cancer Society Research Center, FM & HS, University of Auckland, Private Bag 92019, Auckland 1142, New Zealand; kbishop@auckland.ac.nz

**Keywords:** polyphenols, breast cancer, human trials

## Abstract

Evidence from numerous observational and clinical studies suggest that polyphenolic phytochemicals such as phenolic acids in olive oil, flavonols in tea, chocolate and grapes, and isoflavones in soy products reduce the risk of breast cancer. A dietary food pattern naturally rich in polyphenols is the Mediterranean diet and evidence suggests those of Mediterranean descent have a lower breast cancer incidence. Whilst dietary polyphenols have been the subject of breast cancer risk-reduction, this review will focus on the clinical effects of polyphenols on reducing recurrence. Overall, we recommend breast cancer patients consume a diet naturally high in flavonol polyphenols including tea, vegetables (onion, broccoli), and fruit (apples, citrus). At least five servings of vegetables and fruit daily appear protective. Moderate soy protein consumption (5–10 g daily) and the Mediterranean dietary pattern show the most promise for breast cancer patients. In this review, we present an overview of clinical trials on supplementary polyphenols of dietary patterns rich in polyphenols on breast cancer recurrence, mechanistic data, and novel delivery systems currently being researched.

## 1. Introduction

Breast cancer is the most commonly diagnosed cancer in females worldwide [1]. Diet-related factors are thought to account for around 30% of all cancer in developed countries, with breast cancer being no exception. Obesity, a lack of physical activity, and, to a lesser extent, alcohol increase the risk of breast cancer [2], whereas consumption of vegetables, fruits, legumes, grains, and green tea appear to be protective [3]. In particular, several plant components especially phytochemicals may protect against DNA damage and block specific carcinogen pathways. There are a multitude of in vitro studies outlining the effect specific dietary components have on breast cancer; however, interpretation and clinical application of such studies is problematic, as cell-based studies fail to account for human absorption and metabolism. Presently, there are very few evidence-based nutrition guidelines for breast cancer survivors to follow and many are confused about nutrition support post-diagnosis. Secondary prevention or adjunct therapy through dietary intervention is a cost-effective alternative for preventing the large burden of healthcare associated with breast cancer treatment. In the past decade, epidemiologic and preclinical evidence suggest that polyphenolic phytochemicals present in many plant foods possess chemo-preventive properties against breast cancer [2]. Epidemiological data suggests dietary patterns naturally rich in polyphenols are protective against breast cancer. Whilst data on the nutritional aspect of cancer prevention and the reduction of risk are important, the degree to which the outcomes that can be applied to reducing cancer recurrence is questionable. Increasing evidence suggests that diets providing a variety of polyphenols are useful with regard to breast cancer prevention and cessation.

The health benefits of polyphenols have been linked mostly to their antioxidant effects. Although this is an important contributor, polyphenol phytochemicals also interact with other pathways, especially receptor signalling. Polyphenols have been reported to reduce inflammation and cancer recurrence by (a) acting as an antioxidant or increasing antioxidant gene or protein expression; (b) decreasing cancer cell proliferation; (c) blocking pro-inflammatory cytokines or endotoxin-mediated kinases and transcription factors involved in cancer progression; (d) increasing histone deacetylase activity; or (e) activating transcription factors that antagonize chronic inflammation [4,5]. Polyphenol phytochemicals can interfere with both estrogen receptor (ER) and tyrosine kinase receptor (TKR) signalling, thereby inducing apoptotic and/or autophagy cell death. Estrogen receptors are central to the development of primary and secondary breast cancers. Estrogen binds membrane-initiated steroid signalling (MISS) or TKR to initiate a cascade of effects via estrogen response elements (ERE), AP-1, SP1, and other transcription factors to activate pro-apoptotic genes [6]. There are some indications that polyphenols can bind ER with varying affinities. Thus, it is clear that targeting these ER pathways using dietary polyphenols may affect the development of both primary and secondary breast cancer. The importance of other dietary factors, including meat, fibre, and vitamins, is not yet clear [7]. There has been interest in the potential of naturally occurring cancer chemo-preventive agents, such as polyphenols, to curb the increasing burden of breast cancer treatment [8,9]. Dietary polyphenols may support current medical treatment options to improve prognosis.

This article reviews the current literature on breast cancer clinical trials of polyphenolic phytochemicals with an aim to identify potential nutritional strategies for breast cancer patients, post-diagnosis.

## 2. Methods

The current review discusses the evidence on dietary polyphenols and food patterns naturally high in polyphenols and breast cancer recurrence or relevant biomarkers. In selecting the literature to review, studies that addressed the prognosis and recurrence of breast cancer in survivors were identified. Inclusion criteria included any breast cancer stage and type, human trials only, and intervention commenced after breast cancer diagnosis. Particular attention has been given to human randomised control trials and observational studies on breast cancer survivors. Studies included in Table 1 were human data and must have investigated polyphenol-rich dietary intake or supplements. For inclusion, our definition of a “polyphenol rich diet” were those investigating vegetables (onion, broccoli) and fruit (apples, citrus). Articles from any date of publication or language were considered. PubMed, Google Scholar, and PEN—Practice-Based Nutrition Database—were searched using various key terms, including “breast cancer”, “nutrition”, “polyphenol”, and “human”. Abstracts were reviewed for relevant material.

## 3. Discussion

### 3.1. Dietary Amelioration of Inflammation Associated with Breast Cancer

Many studies suggest that low-grade inflammation is mitigated by healthy dietary habits, such as polyphenols and the Mediterranean food pattern, resulting in lower circulating concentrations of inflammatory markers [17]. Western-type or meat-based patterns are positively associated with low-grade inflammation [18]. Among the components of a healthy diet, whole grains, vegetables and fruits, and fish are all associated with lower inflammation, and a limited number of observational studies suggested a pro-inflammatory action of diets rich in saturated fatty acids or trans-monounsaturated fats [19]. The association between inflammation and cancer has been reported elsewhere [20], citing major mediators nuclear factor kappa B (NF-κB), tumour necrosis factor (TNF), and cyclooxygenase-2 (COX-2), given the combined role in inflammation, cell proliferation, angiogenesis, and metastasis. Inhibition of COX-2 thus blocking the inhibition cascade may be an important mechanism by which polyphenols exert benefit to the breast cancer patient. The consumption of polyphenol-rich foods is thought to have an effect in modulating low-grade inflammation [21].

The inflammatory environment that promotes breast cancer tumour growth links to obesity and metabolic syndrome. Women who gain weight in adulthood and overweight postmenopausal women have a greater risk for breast cancer than lean women [22]. However, there are inconsistencies regarding the effect modification of menopausal status. In contrast, evidence exists showing that overweight and obesity is associated with reduced risk in premenopausal women [23]. Metabolic syndrome (clinically defined as having three of the following factors: Abdominal obesity, hypertension, hyperglycemia, high triglycerides, or low HDL cholesterol [24] has been associated with a 2.6 times higher risk of breast cancer in postmenopausal women [25]. It can be deduced that a range of factors, including age, hormone levels, and obesity and overweight, affect breast cancer risk. Because overweight and obesity are powerful modifiable risk factors [26], interventions, including dietary intervention, should be investigated further. Whilst clear evidence links metabolic syndrome with increased risk of breast cancer, it is also clear that post-diagnosis weight gain occurs in 50%–95% of patients and is associated with poor prognosis. Excess weight gain is associated with elevated inflammatory markers, against which polyphenols may protect.

According to a study conducted on rats, dietary supplementation of a high-fat diet and polyphenols led to dramatic changes in gut microbial community structure [27]. Cranberry polyphenols protected mice on a high-fat, high-sucrose diet against oxidative stress, inflammation, weight gain, and markers of metabolic syndrome [28]. Chronic low-grade inflammation promoted by an individual’s diet and their functioning gut microbiota may influence cancer progression.

Dietary polyphenols may protect against breast cancer progression, despite limited absorption and digestion, raising questions about their mechanism of action. As discussed, polyphenols appear to alter gut microbiota in rats and mice and has also been demonstrated in human studies. It was found that a moderate intake of red wine had positive effects on the composition of the gut microbiota and a reduction in the inflammatory markers [29]. Polyphenols may assist the breast cancer patient by minimizing weight gain, improving the inflammatory profile and altering gut microbiota activity, thus reducing tumour growth.

### 3.2. Antioxidant Action of Polyphenolics

Polyphenols are secondary metabolites of plants and are generally involved in defense against ultraviolet radiation or aggression due to their physiological effects and structure [30]. Many of the biological actions of polyphenols have been attributed to their antioxidant properties; however, recent research has suggested that polyphenols may affect several cellular pathways, thereby exerting a pleiotropic effect [31]. Cellular pathways initiated by polyphenols may delay and reduce the carcinogenic processes in breast tissue [32,33]. Oxidative stress is known to alter the cellular redox status, resulting in altered gene expression by the activation of several redox-sensitive transcription factors. This signaling cascade affects both cell growth and cell death. An increased rate of reactive oxygen species (ROS) production occurs in highly proliferative cancer cells, owing to oncogenic mutations that promote aberrant metabolism. The ability of dietary polyphenols to modulate cellular signal transduction pathways, through the activation or repression of multiple redox-sensitive transcription factors, has been claimed for their potential therapeutic use as chemo-preventive agents [34].

Red wine polyphenols reduce breast cancer cell proliferation in a dose-dependent manner by specifically targeting steroid receptors and modifying the production of ROS [4]. However, it should be noted that it would not be prudent to advise the breast cancer patient to consume alcohol, given the potentially damaging effects. Phenolic phytochemicals have a strong antioxidant potential due to the hydroxyl groups associated with their aromatic rings. Phenolic phytochemicals have been shown to increase the levels of anti-inflammatory genes such as superoxide dismutase (SOD), glutathione peroxidase (GPx), and heme oxygenase (HO)-1 via activation of the transcription factor nuclear factor-erythroid 2 (NF-E2)-related factor 2 (Nrf2). Thus, polyphenols have an inherent capacity to reduce ROS and other free radicals, thereby preventing their activation of oxidative stress and inflammation [35]. Polyphenols are effective free radical scavengers and their antioxidant properties should not be overlooked. In a recent meta-analysis of data from 7500 participants, those who reported a high polyphenol intake, especially of stilbenes and lignans, showed a reduced risk of overall mortality compared to those with lower intakes [36]. Polyphenols where found to be protective against chronic disease, implying a change in oxidative status. The antioxidant properties of polyphenols are thought to delay and to fight the carcinogenic processes in breast tissue [32,33]. Further studies will likely provide additional insights into the mechanism of redox control of breast cancer. Whilst polyphenols appear to reduce oxidative stress, the degree to which breast cancer prognosis is improved is unclear.

### 3.3. Polyphenols Protect DNA from the Carcinogen-Induced Damage

Chronic activation of inflammatory processes is widely regarded as an enabling characteristic towards the development of cancer. We know that chronic inflammation can drive tumour growth and the production of ROS [37]. In turn, ROS can cause DNA damage. Production of ROS, together with deficiencies in the capacity to repair DNA (genotype dependent), can interact to increase carcinogenic capabilities [37,38]. Base-excision repair genes, such as *XRCC1* G399A [37] and *OGG1* C326G, are associated with reduced repair of DNA lesions associated with ROS [39].

The mutagen sensitivity assay (MSA) can be used as a marker of the ability of DNA to respond to and repair DNA damage and hence it has been used to test response to mutagens and bioactives [38]. The Comet and Micronucleus assays have also been extensively used to determine the extent of DNA strand breaks and repair [40,41,42], and there are a number of other methods, including RAD1 focus formation [43], PCR, and the TUNEL assay, as well as numerous others [44].

Germline mutations in DNA mismatch repair genes (*BRCA1*, *BRCA2*, *CHEK2*, *ERCC4*, *FAAP100*, and *TP53BP1*, amongst others) are associated with breast cancer susceptibility [45,46]. In some cases, it may be possible to modify diet to help decrease the risk of breast cancer and breast cancer recurrence [45]. In a study of triple negative breast cancer (TNBC) patients, Lee et al. assessed 16 single nucleotide polymorphisms (SNPs) associated with DNA repair [45]. The authors found that the risk of TNBC was associated with six of the SNPs and that this risk was modified by zinc, folate, and β-carotene levels such that low levels increased risk [45]. These effects were additive. In other studies, it has been reported that high plasma levels of β-carotene, or the consumption of a carotenoid rich diet, were associated with lower levels of breast cancer or breast cancer recurrence [10,11] or a reduction in oxidative stress in those previously treated for breast cancer [47]. Others found that diets rich in fruits and salads, a food pattern traditionally high in polyphenols, was associated with a reduced risk of breast cancer, particularly estrogen and progesterone receptor negative breast cancers [12].

Polyphenols can act as pro- and anti-oxidants, depending on the experimental or environmental conditions [41], and may modify the interaction between carcinogenic capabilities and breast cancer risk. In addition, polyphenols may enhance repair or change methylation status of promoter regions to favour DNA repair, or protect against DNA damage. Adams et al. found that polyphenols from blueberries inhibited cell proliferation and cell migration in human TNBC cell lines [48] and decreased tumour size and inhibited metastasis in a TNBC xenograft study in mice [49]. Similarly, Meeran et al. assessed the effect of Epigallocatechin-3-gallate (EGCG) and sulforaphane, an isothiocynate derived predominantly from plants of the order Brassicales and known to have strong chemo-preventative and anti-inflammatory properties on breast cancer cell lines [50,51]. They found that sulforaphane and EGCG inhibited cell proliferation, telomerase activity, and *human telomerase reverse transcriptase* (*hTERT*) gene expression [50,51]. *hTERT* is widely expressed in cancers, but not in normal cells, and downregulation of *hTERT* in breast cancer can lead to the inhibition of cell proliferation and the induction of apoptosis. Food or dietary compound induced changes in *hTERT* expression, which, in many cases, are due to epigenetic modifications [50,51,52].

### 3.4. Dietary Sources of Polyphenols

Following the systematic search, a small subset of polyphenol types emerged as having human-derived evidence with regard to breast cancer recurrence. This review focuses on the human-derived evidence on breast cancer, and we focus the discussion on phenolic acids, flavonols, and isoflavones. Whilst cell line data on polyphenols such as curcumin and resveratrol are promising, very little has been conducted in human clinical trials.

#### 3.4.1. Phenolic Acids

One of the major dietary sources of dietary phenolic is olive oil, which contains caffeic, oleuropein, and hydroxytyrosol, amongst others. Previous research attributes the health effects of olive oil to its high content in oleic acid. Nowadays, the health benefits of olive oil are also attributed to its phenolic content, namely olepurenoil [53]. Researchers have indicated that the antioxidant capacity of polyphenols in olive oil may reduce the risk of developing cardiovascular diseases and cancer [54].

Studies have indicated that the biological activity of polyphenols in olive oil is higher when they are part of the diet than when these molecules are administered as food supplements [54,55]. The processing of olive oil also determines the variability and availability of polyphenol content in this product. The polyphenol content of olive oil is important, not only for the delivery of compounds with strong anti-oxidant capacity, but also because it exists in conjunction with fatty acids that are potentially oxidised [54]. The phenolic composition of olive oils varies in quantity and quality depending on the olive variety, the age of the tree, and the agricultural techniques used in cultivation.

Recent data suggest a polyphenolic compound found in olive oil, known as oleocanthal, can selectively kill cancerous breast cells while leaving healthy cells intact [56]. Oleocanthal ruptures the lysosome of cancerous cells by inhibiting acid sphingomyelinase activity, which destabilizes the interaction between proteins required for lysosomal membrane stability [56]. The ruptured cell renders the cancer to usual enzymatic degradation and programmed cell death. Further research is needed to confirm findings in human trials, but results are promising. Researchers suggest those on a Mediterranean diet may benefit from the higher consumption of olive oil [56].

Coffee contains numerous compounds, potentially beneficial as well as harmful. With regard to breast cancer, coffee drinking may even have a protective effect. Coffee contains various polyphenols, which inhibit harmful oxidation processes in the body, while the latter include acrylamide, whose high intake in daily diet may have carcinogenic action [57]. In mechanistic cell studies, coffee polyphenols change the expression of STAT5B and ATF-2 modifying cyclin D1 levels in cancer cells [58]. Whilst in vitro studies suggest coffee may offer protection against breast cancer, the overall effect requires clarification, given the paucity of clinical trials.

#### 3.4.2. Flavonols

Flavonols are the major polyphenolic sub-group of flavonoids, which are present in tea, onions, broccoli, and various common fruits. Example polyphenol flavonols include quercetin, kaempferol, myricetin, and isorhamnetin, with an estimated intake of 12.9 mg/day in a typical Western diet [59].

Flavonols may act through anti-oxidant, pro-oxidant, anti-estrogenic, cell signalling pathway modulation, or mitochondrial toxicity to inhibit breast carcinogenesis. One study investigating the effect of flavonols of breast cancer risk reported a risk ratio of 0.94 (0.72, 1.22; *p*-value for test of trend = 0.54) for the sum of flavonol-rich foods. Among the major food sources of flavonols, a significant inverse association with the intake of beans or lentils was reported, but not with tea, onions, apples, string beans, broccoli, green pepper, or blueberries [60]. Despite no overall association between intake of flavonols and risk of breast cancer, there was an inverse association with the intake of beans or lentils. In contrast, a recent meta-analysis of flavonoid intake and breast cancer risk suggested that dietary flavonols and flavones, but not other flavonoid subclasses or total flavonoids, was associated with a decreased risk of breast cancer, especially among post-menopausal women [59]. Given the large range in polyphenols present in the flavonol sub-group, definitive recommendations are difficult; however, it is safe to assume a diet high in beans and legumes and a range of flavonols including onions, apples, citrus, tea, and broccoli are likely to be protective.

#### 3.4.3. Isoflavones

Estrogen is believed to play a role in breast cancer development and progression, and any nutritional intervention that blocks the production or reduces the hormone action is likely to be effective in improving clinical outcomes in breast cancer survivors. Soy food consumption has been attributed to protection against breast cancer, primarily because of the soybean isoflavones (genistein, daidzein, and glycitein), which are natural estrogen receptor modulators. In vitro studies show that genistein inhibits the growth of breast cancer cells, including hormone-dependent and independent cell types at higher concentrations (10–50 µmol/L), while stimulating growth at lower concentrations (<10 µmol/L) [61]. Whilst the structure of soy isoflavones mimics estrogen, the majority of human research fails to detect any clinically relevant estrogenic activity, as determined by estradiol, estrone, and sex hormone binding globulin [62]. In one of the key human intervention studies on soy protein, results were stratified by the amount of soy consumed and showed a dose-response relationship between decreasing risk of breast cancer with an increased soy food intake, translating to a 16% risk reduction per 10 mg of daily isoflavone consumed [63]. However, concerns remain regarding optimal dose of soy foods to ensure improved survival in breast cancer sufferers, and further clinical trials are needed. Soybeans contain a number of anticarcinogens, suggesting that consumption may protect against breast cancer, with non-fermented products such as tofu and soymilk showing more promise.

Unfortunately, clinical outcomes in animal and human epidemiological studies are varied, with 65% of studies reporting no effect or slightly protective against breast cancer risk. A recent review demonstrated the protective effect soy consumption has on breast cancer development, recurrence, and mortality [62]. At this stage, soy phytoestrogens require further research [64]. The protective association of soy food appears more pronounced in postmenopausal women. However, the reduced risk of recurrence results should be interpreted with caution given the modest effect and wide confidence intervals for most studies and the lack of dose response relationship in one positive study.

Both the breast cancer treatment drug Tamoxifen and dietary phytoestrogens bind estrogen receptors, and many have theorised that soy consumption will reduce drug efficacy. In a study on investigating the association of soy food consumption and survival in breast cancer sufferers, women in the highest soy food intake groups had the lowest mortality and recurrence rate compared with women in the lowest intake group, regardless of tamoxifen use. Among women whose soy intake was in the highest quartile, tamoxifen did not confer additional health benefits [65]. Based on this limited epidemiological data, it follows that moderate soy protein consumption (5–10 g/day) in combination with Tamoxifen use represents the optimal treatment combination for relevant breast cancer patients.

Within nutrition science, the critical concept of food synergy recognises that nutrients exist in a purposeful biological sense within foods, delivering them in combinations that reflect biological functionality [66]. Thus, while it is difficult to separate out the effects of foods within a total diet, it is also difficult to study the effects of nutrients and bioactive substances in the isolation of foods [67].

### 3.5. Polyphenol-Rich Dietary Pattern and Breast Cancer Progression

The Mediterranean diet has been shown to reduce body weight by 4.4% over a year and improve the inflammatory profile in cardiac and diabetic groups. Given the tendency for breast cancer survivors to gain weight and risk metabolic syndrome, the Mediterranean diet may assist with weight loss and provide specific benefits over and above the usual low-fat, healthy diet intervention. The Mediterranean diet is a plant-based dietary pattern characterized by a high intake of olive oil, legumes, whole grains, fruit, vegetables, nuts, seeds, fish, and is rich in dietary polyphenols. The diet has been linked to a decreased risk of developing breast cancer [48]. The Mediterranean diet contains a wide range of various polyphenols, particularly from nuts, fruit, and coffee [68], and represents a potential population approach to increasing the intake of polyphenols. Epidemiological evidence strongly suggests that long-term consumption of diets rich in plant polyphenols, much like that of the Mediterranean diet, can offer protection against development of major chronic and neurodegenerative diseases [69,70]. Suggested mechanisms through which the Mediterranean diet may impact breast cancer initiation and proliferation include increased insulin sensitivity and reduction of excess insulin production, anti-inflammatory and antioxidant effects of the diet, high fibre content, and an association with reduced risk of excess weight gain and obesity [48]. The health benefits of the Mediterranean diet are likely a synergistic effect of weight loss, polyphenol intake, and improved glycemic control.

There are three main randomised trials investigating the effect of following a Mediterranean diet pattern and the prognosis following treatment for breast cancer. The results, however, are mixed. In 2007, The Women’s Healthy Eating and Living (WHEL) Randomised Trial found that a diet high in vegetable, fruit, and fibre and low in fat intake did not reduce additional breast cancer events or mortality over a relatively long follow-up period [13]. These results are at odds with the Women’s Intervention Nutrition Study (WINS), a randomised trial that focused on a low-fat diet and weight loss, reporting that this diet was associated with longer relapse-free survival of breast cancer patients [71]. Follow up times and differences in menopausal status between studies may explain outcomes. Difficulties in ascertaining the polyphenol content of these diets make conclusions regarding efficacy difficult.

Another reason for the difference in the results of these trials may be that, in WINS, the women lost weight in the randomised group, whereas those women in the WHEL study had an iso-caloric diet by design, and the women in the intervention group gained around 1 kg. The results from previous observational studies suggesting calorie reduction and weight loss are beneficial in breast cancer prognosis may add context to this situation and show why the results of the WHEL study were to no effect. Such an interpretation is verified by the relatively consistent observations that overweight and obese breast cancer patients have a worse prognosis than lean patients [1,72,73,74]. The Mediterranean diet has been shown to support weight loss in participants and as such may offer multiple benefits in polyphenol intake and weight loss.

The most recent randomised trial investigating the effects of a Mediterranean macrobiotic lifestyle on breast cancer prognosis is the DIANA-5 trial [75]. It demonstrated that dietary modification based on Mediterranean and macrobiotic dietary principles can reduce body weight, and the bioavailability of sex hormones and growth factors may promote tumour growth [76,77]. The diet consisted of low consumption of fats, refined carbohydrates and animal products, and the high consumption of whole grain cereals, legumes, and vegetables.

Chemotherapy works to significantly decrease recurrences and improve survival in women with early breast cancer, but a major side effect is weight gain which, as discussed, is associated with a poorer prognosis [78]. The trial showed this specific diet significantly decreases body weight and waist circumference, thereby improving insulin sensitivity [79]. Like the WINS trial, only post-menopausal women were included in the study. The results may have differed for pre-menopausal women if they were also included.

Overall, the DIANA-5 trial has the potential to provide a clear answer to the hypothesis that a comprehensive modification of diet can lead to a longer event-free survival among women after breast cancer treatment [75]. Intervention has been shown to be effective in changing lifestyle in terms of diet and weight loss. Combined with other modifiable factors, a Mediterranean diet that focuses on weight loss and reducing insulin resistance may have substantial benefits for women previously treated for breast cancer. All of these studies have pieced together components that warrant further investigation to the role of a Mediterranean-based diet and breast cancer prognosis, event-free survival, and mortality.

### 3.6. Disease Characteristics and Biomarkers

A reduction in breast cancer incidence and mortality is the gold standard criteria for success in a clinical trial; however, this approach is expensive and ethically difficult to implement. The use of surrogate breast cancer biomarkers is an appealing alternative. Breast cancer biomarkers useful for investigating the efficacy of polyphenols include specific oncogenic pathways (e.g., COX-2, or prostaglandin E2, a product of COX mediated catalysis), levels of circulating disease related proteins, such as ostrodial or estrogen, changes in breast cancer histology and cytology, genomic alterations

A major challenge in the treatment of breast cancer is its high heterogeneity from patient to patient, which initiated its classification into three major molecular subtypes—estrogen receptors (ER), progesterone receptors (PR), and HER2, hormone receptor positive with luminal A (ER+PR+HER2−) and luminal B (ER+PR+HER2+) phenotypes, HER2 positive (ER−PR−HER2+), and triple negative/basal-like (ER−PR−HER2−) [80,81,82]. About 70% of breast cancers are estrogen receptor positive [83]. Recent data suggest that molecular subtypes differ substantially in the intracellular pathways responsible for cell growth and metastatic spread, suggesting a wide array of potential molecular targets of polyphenols [84]. The efficacy of polyphenolic therapy is likely to differ pending the breast cancer stage and subtype.

### 3.7. Epigenetic Potential of Polyphenolic Phytochemicals

Epigenetics refers to heritable changes in DNA that are involved in the control of gene expression. Epigenetic mechanisms include changes in DNA methylation, histone modification, and non-coding RNAs [84]. While epigenetic characteristics are sometimes inherited they can also be modified by environmental and dietary factors. Inflammatory pathways can trigger epigenetic switches from nontransformed to metastatic cancer cells via signalling involving NF κB and STAT3 transcription factors, microRNAs (Lin28 and let-7), and IL-6 cytokines [85]. Moreover, the polyphenols resveratrol and quercetin decreased miRNA-155 and inhibited NF-κB-involved inflammation in a cancer cell line study. Increasing evidence suggests polyphenols are capable of influencing epigenetic characteristics relevant to cancer progression. It is beyond the scope of this review to outline all the research of all aspects of the epigenetic potential of polyphenols; other reviews have been completed [85]. Of the more notable epigenetic modification by polyphenols, epigenetically modified genes can be restored, inactivated methylated genes can be demethylated, and histone complexes can be rendered transcriptionally active by dietary intervention. Common to cancer initiation is the inhibition of tumour suppressor genes by DNA methylation of transcription factors. DNA methyltransferase (DNMT) inhibitors can undergo such methylation, which polyphenols have been demonstrated to reverse [86].

Polyphenols can also alter heritable gene expression, activity of epigenetic machinery and decreases micro-RNAs related to inflammation and cancer growth. So far, it is not clear whether the occasional or typical dietary intake of polyphenols results in long-term epigenetic regulation of gene expression, downstream chemo-preventative effects, or both.

### 3.8. Bioavailability of Polyphenols

Biological properties of polyphenols depend on their bioavailability. The chemical structure of polyphenols determines their rate and extent of intestinal absorption, as well as the nature of the metabolites circulating in the plasma. For most flavonoids absorbed in the small intestine, the plasma concentration rapidly decreases (elimination half-life period of 1–2 h). The elimination half-life period for quercetin is much higher (24 h) probably due to its particularly high affinity for plasma albumin [87]. Flavonols, isoflavones, flavones, and anthocyanins are usually glycosylated. Following high-dose polyphenol administration, metabolism occurs primarily in the liver, whereas, when smaller doses were administered, metabolism took place first at the intestinal mucosa, the liver playing a secondary role to further modify the conjugated polyphenol. This implies that the intestine is an important site for metabolism of food-derived polyphenols [88]. Intestinal microbiological fermentation decreases the bioavailability of the many polyphenols; however, it also gives rise to metabolites that may be more bioactive than the native polyphenols [88]. Metabolic responses based on dose also suggest that any potential benefit will vary based on the polyphenol dose used. Studies on ideal dose and delivery route are needed.

To circumvent poor bioavailability of polyphenols, a current area of promising research is using nanotechnology. One such nanotechnology, titled “Nano emulsions”, are a class of extremely small droplets that allow polyphenol phytochemicals to be transported through the cell membranes more easily, resulting in an increased concentration in plasma and improved bioavailability. Curcumin Nano emulsions show 85% inhibition of 12-*O*-Tetradecanoylphorbol-13-acetate (TPA)-induced mouse ear inflammation as well as the inhibition of cyclin D1 expression. In addition, dibenzoylmethane (DBM) Nano emulsions improve oral bioavailability of curcumin 3-fold, compared with the conventional DBM emulsions [89]. The degree to which improved bioavailability improves survival in breast cancer patients is still to be determined, as there is likely a dose-response that is still to be ascertained.

### 3.9. Limitations (Toxicity, Bioavailability, Challenges and Weaknesses Associated with Human Trials, etc.)

Several factors have been proposed to explain differences observed between the positive effects of polyphenol consumption reported in epidemiological studies and the unclear to negative findings reported in intervention trials with supplements. These factors include the following: (1) differing doses of administered compounds; (2) additive or synergistic effects, such as those between polyphenols and other antioxidants, present in whole foods but not in supplements; and (3) differences in bioavailability and metabolism [88]. Results from randomised clinical trials vary to those of in vitro studies largely as a result of these factors.

With any human dietary study, interpreting outcomes and defining appropriate dietary recommendations can be extremely difficult. Studies typically involve many methodological considerations such as dietary pattern differences across populations, accurately measuring food intake, biological mechanisms, genetic variations, food definitions, bias, and other confounding factors [90]. Adding further complication is that many studies between cancer and diet provide weak associations, whereby confounding factors, exposure misclassification, and other biases, even modest ones, can have a large impact on the overall conclusions [91]. To best answer questions regarding efficacy of dietary polyphenols, in vitro studies of polyphenol metabolites should be followed up with human clinical trials and we would recommend that further studies use placebo controlled, double-blind trials that extend over many years with a sufficient sample size. Unfortunately, such studies are expensive to conduct.

## 4. Conclusions

Whilst recognizing the broad nature of investigating the efficacy of polyphenols for breast cancer patients, we can conclude the following based on clinical and observational studies. Early diagnosed breast cancer patients should consume at least five servings of vegetables and fruit, and we recommend those high in flavonols such as onions, broccoli, apples, and citrus, amongst others. Both green and black tea consumption is protective, with 3+ cups of green tea being particularly helpful. We would recommend women diagnosed with breast cancer to adopt a moderate soy protein consumption (5–10 g/day) from non-fermented soy products such as soymilk and tofu.

The Mediterranean diet appears useful in assisting with weight control and improving metabolic syndrome. It is a dietary pattern naturally high in legumes and olive oil, both of which have been independently reported to improve in vitro and in vivo breast cancer recurrence and biomarkers of disease. Foods rich in polyphenols are the preferred methods of delivery over supplements, until more is known. Further research should include specific dietary foods in large randomized control trials, which, the authors recognize, are expensive to conduct.

## Figures and Tables

**Table 1:** Clinical studies of polyphenols in breast cancer patients. Table includes human studies only and those with a dietary or supplemental intervention. Abbreviations: BCa—Breast cancer.

Author, Year	Research Design/Assessment/Outcome Measure	Participants	Summary Outcome
Rock, Natarajan et al., 2009 [10]	Design; Observational. Assessed intake of vegetables, fruit and fibre. Outcome: Time to secondary BCa cancer event	3043 early-mid diagnosed BCa patients	Greater intake of fruit and vegetables naturally high in polyphenols and carotenoids, was associated with improved likelihood of breast cancer–free survival regardless of study group assignment. HR = 0.67
Mignone, Giovannucci et al., 2009 [11]	Design: Observational. Assessed dietary intake of fruit and vegetable consumption. Outcome: risk of breast cancer	5707 BCa patients; 6389 Controls	A high consumption of fruit and vegetables naturally high in polyphenols and carotenoids may reduce the risk of premenopausal but not postmenopausal breast cancer, particularly among smokers
Baglietto, Krishnan et al., 2011 [12]	Design: Observational. Assessed dietary intake patterns. Outcome: Risk of invasive breast cancer	20,967 women of which 815 develop invasive BCa	A dietary pattern rich in fruit and salad might protect against invasive breast cancer and that the effect might be stronger for ER- and PR-negative tumours
Pierce, J.P., Natarajan, L., Caan, B.J. et al., 2007 [13]	Design: Intervention Education to promote 5 servings of fruit and vegetable. Outcome: Time to secondary BCa event	1537 Bca patients; 1551 controls	Among survivors of early stage breast cancer, adoption of a diet that was very high in vegetables, fruit, and fibre and low in fat did not reduce additional breast cancer events or mortality during a 7.3-year follow-up period. Unfortunately, the control group also received written education material
Sartippour M.R., Rao J.Y., Apple S., Wu et al., 2004 [14]	Design: Intervention. 200 mg isoflavones for 2-weeks. Assessment: Direct breast tissue samples from patients were assessed for cancer growth	17 BCa patients; 26 Controls	No change in apoptosis/mitosis ratio
DiSilvestro R.A., Goodman, J., Dy, E., Lavalle, G. 2005 [15]	Design: Intervention. 138 mg isoflavones for 24-days. Assessment: Blood samples were assessed for oxidative status	7 BCa patients, crossover design	Increased SOD activity. No change in oxidative stress markers
Inoue, M., Tajima, K., Mizutani, M. et al., 2001 [16]	Design: Observational. Assessment: Consumption of green tea	1160 women of which 133 develop BCa	3+ cups of green tea daily was associated with lower BCa recurrence in early stages (HR = 0.69, 95% Cl 0.47–1.00)

## References

[1] Hauner H., Hauner D. (2010). The Impact of Nutrition on the Development and Prognosis of Breast Cancer. Breast Care (Basel).

[2] Key T.J., Allen N.E., Spencer E.A., Travis R.C. (2002). The effect of diet on risk of cancer. Lancet.

[3] Gandini S., Merzenich H., Robertson C., Boyle P. (2000). Meta-analysis of studies on breast cancer risk and diet: The role of fruit and vegetable consumption and the intake of associated micronutrients. Eur. J. Cancer.

[4] Damianaki A., Bakogeorgou E., Kampa M., Notas G., Hatzoglou A., Panagiotou S., Gemetzi C., Kouroumalis E., Martin P.M., Castanas E. (2000). Potent inhibitory action of red wine polyphenols on human breast cancer cells. J. Cell. Biochem..

[5] Williamson G., Manach C. (2005). Bioavailability and bioefficacy of polyphenols in humans. II. Review of 93 intervention studies. Am. J. Clin. Nutr..

[6] Aiyer H.S., Warri A.M., Woode D.R., Hilakivi-Clarke L., Clarke R. (2012). Influence of berry polyphenols on receptor signaling and cell-death pathways: Implications for breast cancer prevention. J. Agric. Food Chem..

[7] Abdulla M., Gruber P. (2000). Role of diet modification in cancer prevention. Biofactors.

[8] Lambert J.D., Yang C.S. (2003). Mechanisms of cancer prevention by tea constituents. J. Nutr..

[9] Spagnuolo C., Russo G.L., Orhan I.E., Habtemariam S., Daglia M., Sureda A., Nabavi S.F., Devi K.P., Loizzo M.R., Tundis R. (2015). Genistein and cancer: Current status, challenges, and future directions. Adv. Nutr..

[10] Rock C.L., Natarajan L., Pu M., Thomson C.A., Flatt S.W., Caan B.J., Gold E.B., Al-Delaimy W.K., Newman V.A., Hajek R.A. (2009). Longitudinal biological exposure to carotenoids is associated with breast cancer-free survival in the Women’s Healthy Eating and Living Study. Cancer Epidemiol. Biomark. Prev..

[11] Mignone L.I., Giovannucci E., Newcomb P.A., Titus-Ernstoff L., Trentham-Dietz A., Hampton J.M., Willet W.C., Egan K.M. (2009). Dietary carotenoids and the risk of invasive breast cancer. Int. J. Cancer.

[12] Baglietto L., Krishnan K., Severi G., Hodge A., Brinkman M., English D.R., McLean C., Hopper J.L., Giles G.G. (2011). Dietary patterns and risk of breast cancer. Br. J. Cancer.

[13] Pierce J.P., Natarajan L., Caan B.J., Parker B.A., Greenberg E.R., Flatt S.W., Rock C.L., Kealey S., Al-Delaimy W.K., Bardwell W.A. (2007). Influence of a diet very high in vegetables, fruit, and fiber and low in fat on prognosis following treatment for breast cancer: The Women’s Healthy Eating and Living (WHEL) randomized trial. JAMA.

[14] Sartippour M.R., Rao J.Y., Apple S., Wu D., Henning S., Wang H., Elashoff R., Rubio R., Heber D., Brooks M.N. (2004). A pilot clinical study of short-term isoflavone supplements in breast cancer patients. Nutr. Cancer.

[15] DiSilvestro R.A., Goodman J., Dy E., Lavalle G. (2005). Soy isoflavone supplementation elevates erythrocyte superoxide dismutase, but not plasma ceruloplasmin in postmenopausal breast cancer survivors. Breast Cancer Res. Treat..

[16] Inoue M., Tajima K., Mizutani M., Iwata H., Iwase T., Miura S., Hirose K., Hamajima N., Tominaga S. (2001). Regular consumption of green tea and the risk of breast cancer recurrence: Follow-up study from the Hospital-based Epidemiologic Research Program at Aichi Cancer Center (HERPACC), Japan. Cancer Lett..

[17] Centritto F., Iacoviello L., di Giuseppe R., De Curtis A., Costanzo S., Zito F., Grioni S., Sieri S., Donati M.B., de Gaetano G. (2009). Dietary patterns, cardiovascular risk factors and C-reactive protein in a healthy Italian population. Nutr. Metab. Cardiovasc. Dis..

[18] Barbaresko J., Koch M., Schulze M.B., Nothlings U. (2013). Dietary pattern analysis and biomarkers of low-grade inflammation: A systematic literature review. Nutr. Rev..

[19] Calder P.C., Ahluwalia N., Brouns F., Buetler T., Clement K., Cunningham K., Esposito K., Jonsson L.S., Kolb H., Lansink M. (2011). Dietary factors and low-grade inflammation in relation to overweight and obesity. Br. J. Nutr..

[20] Ramos S. (2008). Cancer chemoprevention and chemotherapy: Dietary polyphenols and signalling pathways. Mol. Nutr. Food Res..

[21] Bonaccio M., Pounis G., Cerletti C., Donati M.B., Iacoviello L., de Gaetano G. (2016). Mediterranean diet, dietary polyphenols and low-grade inflammation: Results from the moli-sani study. Br. J. Clin. Pharmacol..

[22] Lahmann P.H., Schulz M., Hoffmann K., Boeing H., Tjonneland A., Olsen A., Overvad K., Key T.J., Allen N.E., Khaw K.T. (2005). Long-term weight change and breast cancer risk: The European prospective investigation into cancer and nutrition (EPIC). Br. J. Cancer.

[23] National Cancer Institute United States of America: Obesity and Cancer Risk, 2012. http://www.cancer.gov/about-cancer/causes-prevention/risk/obesity/obesity-fact-sheet.

[24] Expert Panel on Detection, Evaluation and Treatment of High Cholesterol in Adults (2001). Executive Summary of the Third Report of the National Cholesterol Education Program (NCEP) Expert Panel on Detection, Evaluation and Treatment of High Blood Cholesterol in Adults (Adult Treatment Panel III). J. Am. Med. Assoc..

[25] Agnoli C., Berrino F., Abagnato C.A., Muti P., Panico S., Crosignani P., Krogh V. (2010). Metabolic syndrome and postmenopausal breast cancer in the ORDET cohort: A nested case-control study. Nutr. Metab. Cardiovasc. Dis..

[26] Chan D.S., Vieira A.R., Aune D., Bandera E.V., Greenwood D.C., McTiernan A., Navarro-Rosenblatt D., Thune I., Vieira R., Norat T. (2014). Body mass index and survival in women with breast cancer-systematic literature review and meta-analysis of 82 follow-up studies. Ann. Oncol..

[27] Roopchand D.E., Carmody R.N., Kuhn P., Moskal K., Rojas-Silva P., Turnbaugh P.J., Raskin I. (2015). Dietary Polyphenols Promote Growth of the Gut Bacterium Akkermansia muciniphila and Attenuate High-Fat Diet-Induced Metabolic Syndrome. Diabetes.

[28] Anhe F.F., Roy D., Pilon G., Dudonne S., Matamoros S., Varin T.V., Garofalo C., Moine Q., Desjardins Y., Levy E. (2015). A polyphenol-rich cranberry extract protects from diet-induced obesity, insulin resistance and intestinal inflammation in association with increased *Akkermansia* spp. population in the gut microbiota of mice. Gut.

[29] Moreno-Indias I., Sanchez-Alcoholado L., Perez-Martinez P., Andres-Lacueva C., Cardona F., Tinahones F., Queipo-Ortuno M.I. (2016). Red wine polyphenols modulate fecal microbiota and reduce markers of the metabolic syndrome in obese patients. Food Funct..

[30] Ramos S. (2007). Effects of dietary flavonoids on apoptotic pathways related to cancer chemoprevention. J. Nutr. Biochem..

[31] Maraldi T., Vauzour D., Angeloni C. (2014). Dietary polyphenols and their effects on cell biochemistry and pathophysiology 2013. Oxid. Med. Cell. Longev..

[32] Varinska L., Gal P., Mojzisova G., Mirossay L., Mojzis J. (2015). Soy and breast cancer: Focus on angiogenesis. Int. J. Mol. Sci..

[33] Yang C.S., Lambert J.D., Sang S. (2009). Antioxidative and anti-carcinogenic activities of tea polyphenols. Arch. Toxicol..

[34] Di Domenico F., Foppoli C., Coccia R., Perluigi M. (2012). Antioxidants in cervical cancer: Chemopreventive and chemotherapeutic effects of polyphenols. Biochim. Biophys. Acta.

[35] Rahman I., Biswas S.K., Kirkham P.A. (2006). Regulation of inflammation and redox signaling by dietary polyphenols. Biochem. Pharmacol..

[36] Duell E.J., Millikan R.C., Pittman G.S., Winkel S., Lunn R.M., Tse C.K., Eaton A., Mohrenweiser H.W., Newman B., Bell D.A. (2001). Polymorphisms in the DNA repair gene XRCC1 and breast cancer. Cancer Epidemiol. Biomark. Prev..

[37] Tresserra-Rimbau A., Rimm E.B., Medina-Remon A., Martinez-Gonzalez M.A., Lopex-Sabeter M.C., Covas M.I., Corella D., Salas-Salvado J., Gomez-Gracia E., Lapetra J. (2014). Polyphenol intake and mortality risk: A re-analysis of the PREDIMED trial. BMC Med..

[38] Kosti O., Byrne C., Meeker K.L., Watkins K.M., Loffredo C.A., Shields P.G., Schwartz M.D., Willey S.C., Cocilovo C., Zheng Y.L. (2010). Mutagen sensitivity, tobacco smoking and breast cancer risk: A case-control study. Carcinogenesis.

[39] Songserm N., Promthet S., Pientong C., Ekalaksananan T., Chopjitt P., Wiangnon S. (2014). Gene-environment interaction involved in cholangiocarcinoma in the Thai population: Polymorphisms of DNA repair genes, smoking and use of alcohol. BMJ Open.

[40] Collins A.R. (2004). The comet assay for DNA damage and repair: Principles, applications, and limitations. Mol. Biotechnol..

[41] Rodeiro I., Delgado R., Garrido G. (2014). Effects of a Mangifera indica L. stem bark extract and mangiferin on radiation-induced DNA damage in human lymphocytes and lymphoblastoid cells. Cell Prolif..

[42] Bishop K.S., Erdrich S., Karunasinghe N., Han D.Y., Zhu S., Jesuthasan A., Ferguson L.R. (2015). An investigation into the association between DNA damage and dietary fatty acid in men with prostate cancer. Nutrients.

[43] Powell S.N., Riaz N., Mutter W., Ng C.K.Y., Delsite R., Piscuoglio S., King T.A., Martelotto L., Sakr R., Brogi E. (2016). Abstract S4–03: A functional assay for homologous recombination (HR) DNA repair and whole exome sequencing reveal that HR-defective sporadic breast cancers are enriched for genetic alterations in DNA repair genes. Cancer Res..

[44] Kumari S., Rastogi R., Singh K., Singh S., Sinha R. (2008). DNA damage: Detection strategies. EXCLI J..

[45] Lee E., Levine E.A., Franco V.I., Allen G.O., Gong F., Zhang Y., Hu J.J. (2014). Combined genetic and nutritional risk models of triple negative breast cancer. Nutr. Cancer.

[46] Campeau P.M., Foulkes W.D., Tischkowitz M.D. (2008). Hereditary breast cancer: New genetic developments, new therapeutic avenues. Hum. Genet..

[47] Thomson C.A., Stendell-Hollis N.R., Rock C.L., Cussler E.C., Flatt S.W., Pierce J.P. (2007). Plasma and dietary carotenoids are associated with reduced oxidative stress in women previously treated for breast cancer. Cancer Epidemiol. Biomark. Prev..

[48] Adams L.S., Phung S., Yee N., Seeram N.P., Li L., Chen S. (2010). Blueberry phytochemicals inhibit growth and metastatic potential of MDA-MB-231 breast cancer cells through modulation of the phosphatidylinositol 3-kinase pathway. Cancer Res..

[49] Adams L.S., Kanaya N., Phung S., Liu Z., Chen S. (2011). Whole blueberry powder modulates the growth and metastasis of MDA-MB-231 triple negative breast tumors in nude mice. J. Nutr..

[50] Meeran S.M., Patel S.N., Tollefsbol T.O. (2010). Sulforaphane causes epigenetic repression of hTERT expression in human breast cancer cell lines. PLoS ONE.

[51] Meeran S.M., Patel S.N., Chan T.H., Tollefsbol T.O. (2011). A novel prodrug of epigallocatechin-3-gallate: Differential epigenetic hTERT repression in human breast cancer cells. Cancer Prev. Res. (Phila.).

[52] Meeran S.M., Patel S.N., Li Y., Shukla S., Tollefsbol T.O. (2012). Bioactive dietary supplements reactivate ER expression in ER-negative breast cancer cells by active chromatin modifications. PLoS ONE.

[53] Martin-Pelaez S., Covas M.I., Fito M., Kusar A., Pravst I. (2013). Health effects of olive oil polyphenols: Recent advances and possibilities for the use of health claims. Mol. Nutr. Food Res..

[54] De la Torre-Robles A., Rivas A., Lorenzo-Tovar M.L., Monteagudo C., Mariscal-Arcas M., Olea-Serrano F. (2014). Estimation of the intake of phenol compounds from virgin olive oil of a population from southern Spain. Food Addit. Contam. Part A Chem. Anal. Control Expo. Risk Assess..

[55] Covas M.I., Nyyssonen K., Poulsen H.E., Kaikkonen J., Zunft H.J., Kiesewetter H., Gaddi A., de la Torre R., Mursu J., Baumler H. (2006). The effect of polyphenols in olive oil on heart disease risk factors: A randomized trial. Ann. Intern. Med..

[56] LeGendre O., Breslin P.A., Foster D.A. (2015). (−)-Oleocanthal rapidly and selectively induces cancer cell death via lysosomal membrane permeabilization. Mol. Cell. Oncol..

[57] Wierzejska R. (2015). Coffee consumption vs. cancer risk—A review of scientific data. Rocz. Panstwowego Zakladu Hig..

[58] Oleaga C., Ciudad C.J., Noe V., Izquierdo-Pulido M. (2012). Coffee polyphenols change the expression of STAT5B and ATF-2 modifying cyclin D1 levels in cancer cells. Oxid. Med. Cell. Longev..

[59] Cui L., Liu X., Tian Y., Xie C., Li Q., Cui H., Sun C. (2013). Flavonoids, flavonoid subclasses and breast cancer risk: A meta-analysis of epidemiologic studies. PLoS ONE.

[60] Adebamowo C.A., Cho E., Sampson L., Katan M.B., Spiegelman D., Willett W.C., Holmes M.D. (2005). Dietary flavonols and flavonol-rich foods intake and the risk of breast cancer. Int. J. Cancer.

[61] Constantinou A., Huberman E. (1995). Genistein as an inducer of tumor cell differentiation: Possible mechanisms of action. Proc. Soc. Exp. Biol. Med..

[62] Fritz H., Seely D., Flower G., Skidmore B., Fernandes R., Vadeboncoeur S., Kennedy D., Cooley K., Wong R., Sagar S. (2013). Soy, red clover, and isoflavones and breast cancer: A systematic review. PLoS ONE.

[63] Shike M., Doane A.S., Russo L., Cabal R., Reis-Filho J.S., Gerald W., Cody H., Khanin R., Bromberg J., Norton L. (2014). The effects of soy supplementation on gene expression in breast cancer: A randomized placebo-controlled study. J. Natl. Cancer Inst..

[64] Messina M.J., Persky V., Setchell K.D., Barnes S. (1994). Soy intake and cancer risk: A review of the in vitro and in vivo data. Nutr. Cancer.

[65] Shu X.O., Zheng Y., Cai H., Gu K., Chen Z., Zheng W., Lu W. (2009). Soy food intake and breast cancer survival. JAMA.

[66] Jacobs D.R., Gross M.D., Tapsell L.C. (2009). Food synergy: An operational concept for understanding nutrition. Am. J. Clin. Nutr..

[67] Tapsell L.C. (2014). Foods and food components in the Mediterranean diet: Supporting overall effects. BMC Med..

[68] Saura-Calixto F., Goni I. (2006). Antioxidant capacity of the Spanish Mediterranean Diet. Food Chem..

[69] Arts I.C., Hollman P.C. (2005). Polyphenols and disease risk in epidemiologic studies. Am. J. Clin. Nutr..

[70] Scalbert A., Manach C., Morand C., Remesy C., Jimenez L. (2005). Dietary polyphenols and the prevention of diseases. Crit. Rev. Food Sci. Nutr..

[71] Chlebowski R.T., Blackburn G.L., Thomson C.A., Nixon D.W., Shapiro A., Hoy M.K., Goodman M.T., Giuliano A.E., Karanja N., McAndrew P. (2006). Dietary fat reduction and breast cancer outcome: Interim efficacy results from the Women’s Intervention Nutrition Study. J. Natl. Cancer Inst..

[72] Hauner D., Hauner H. (2014). Metabolic syndrome and breast cancer: Is there a link?. Breast Care (Basel).

[73] McTiernan A., Irwin M., Vongruenigen V. (2010). Weight, physical activity, diet, and prognosis in breast and gynecologic cancers. J. Clin. Oncol..

[74] Protani M., Coory M., Martin J.H. (2010). Effect of obesity on survival of women with breast cancer: Systematic review and meta-analysis. Breast Cancer Res. Treat..

[75] Villarini A., Pasanisi P., Traina A., Mano M.P., Bonanni B., Panico S., Scipioni C., Galasso R., Paduos A., Simeoni M. (2012). Lifestyle and breast cancer recurrences: The DIANA-5 trial. Tumori.

[76] Berrino F., Bellati C., Secreto G., Camerini E., Pala V., Panico S., Allegro G., Kaaks R. (2001). Reducing bioavailable sex hormones through a comprehensive change in diet: The diet and androgens (DIANA) randomized trial. Cancer Epidemiol. Biomark. Prev..

[77] Berrino F., Villarini A., De Petris M., Raimondi M., Pasanisi P. (2006). Adjuvant diet to improve hormonal and metabolic factors affecting breast cancer prognosis. Ann. N. Y. Acad. Sci..

[78] Villarini A., Pasanisi P., Raimondi M., Gargano G., Bruno E., Morelli D., Evangelista A., Curtosi P., Berrino F. (2012). Preventing weight gain during adjuvant chemotherapy for breast cancer: A dietary intervention study. Breast Cancer Res. Treat..

[79] Dahabreh I.J., Linardou H., Siannis F., Fountzilas G., Murray S. (2008). Trastuzumab in the adjuvant treatment of early-stage breast cancer: A systematic review and meta-analysis of randomized controlled trials. Oncologist.

[80] Engstrom M.J., Opdahl S., Hagen A.I., Romundstad P.R., Akslen L.A., Haugen O.A., Vatten L.J., Bofin A.M. (2013). Molecular subtypes, histopathological grade and survival in a historic cohort of breast cancer patients. Breast Cancer Res. Treat..

[81] Schnitt S.J. (2010). Classification and prognosis of invasive breast cancer: From morphology to molecular taxonomy. Mod. Pathol..

[82] Staaf J., Ringner M. (2014). Making breast cancer molecular subtypes robust?. J. Natl. Cancer Inst..

[83] Jonat W., Pritchard K.I., Sainsbury R., Klijn J.G. (2006). Trends in endocrine therapy and chemotherapy for early breast cancer: A focus on the premenopausal patient. J. Cancer Res. Clin. Oncol..

[84] Jenkins E.O., Deal A.M., Anders C.K., Prat A., Perou C.M., Carey L.A., Muss H.B. (2014). Age-specific changes in intrinsic breast cancer subtypes: A focus on older women. Oncologist.

[85] Vanden Berghe W. (2012). Epigenetic impact of dietary polyphenols in cancer chemoprevention: Lifelong remodeling of our epigenomes. Pharmacol. Res..

[86] Link A., Balaguer F., Shen Y., Lozano J.J., Leung H.C., Boland C.R., Goel A. (2013). Curcumin modulates DNA methylation in colorectal cancer cells. PLoS ONE.

[87] Tapiero H., Tew K.D., Ba G.N., Mathe G. (2002). Polyphenols: Do they play a role in the prevention of human pathologies?. Biomed. Pharmacother..

[88] Bohn T. (2014). Dietary factors affecting polyphenol bioavailability. Nutr. Rev..

[89] Huang Q., Yu H., Ru Q. (2010). Bioavailability and delivery of nutraceuticals using nanotechnology. J. Food Sci..

[90] Miller P.E., Alexander D.D., Weed D.L. (2014). Uncertainty of results in nutritional epidemiology. Nutr. Today.

[91] Alexander D.D., Weed D.L., Miller P.E., Mohamed M.A. (2015). Red Meat and Colorectal Cancer: A Quantitative Update on the State of the Epidemiologic Science. J. Am. Coll. Nutr..

